# Effect of LongZhang Gargle on Dual-Species Biofilm of Candida albicans and Streptococcus mutans

**DOI:** 10.1155/2021/6654793

**Published:** 2021-03-22

**Authors:** Jinglei Gong, Die Hu, Jinzhi He, Ling Zou, Zhu Chen, Mingyun Li

**Affiliations:** ^1^State Key Laboratory of Oral Diseases, West China Hospital of Stomatology, Sichuan University, No.14, 3rd Section of South RenMin Rd, Chengdu, Sichuan 610041, China; ^2^Department of Operative Dentistry and Endodontics, West China Hospital of Stomatology, Sichuan University, Chengdu, Sichuan, China; ^3^Guiyang Hospital of Stomatology, Zunyi Medical University, Guiyang 550002, China

## Abstract

Bioactive natural products have become a hot spot for oral disease treatments. At the present study, LongZhang Gargle was investigated for its effects on single-species biofilms of *Candida albicans* and dual-species biofilms of *Candida albicans* and *Streptococcus mutans*. Two different models of single and dual-species biofilms were grown in YNBB medium under appropriate conditions. Biofilm biomass, biofilm architecture, and cell activity in biofilms were assessed using Crystal Violet Staining, MTT, scanning electron microscopy (SEM), and confocal laser scanning microscopy (CLSM). Significant reductions of biofilm biomass and fungus activity were obtained when treated with LongZhang Gargle at 2% (*P* < 0.05), 4% (*P* < 0.05), and 8% (*P* < 0.05) in single-species biofilms of *C. albicans*, and at 4% (*P* < 0.05) and 8% (*P* < 0.05) in double-species biofilms. Suppression of density, thickness, and the proportion of hyphae and fungal spores were obtained under SEM and CLSM. In conclusion, LongZhang Gargle affects single and dual-species biofilms by inhibiting biofilm biomass, cell activity, and formation of hyphae, but it does not affect the production of Extracellular polysaccharides (EPS). We speculate that LongZhang Gargle would be a promising natural drug, which can be used in treatment against *C. albicans* and *S. mutans* in oral diseases.

## 1. Introduction

Because of the particularity of the environment, the oral is suitable for the growth of microorganisms, leading to many diseases [1]. *Candida albicans* (*C. albicans*) is a common fungus species parasite in the oral cavity [2]. When the body's immune ability declines or the toxicity of bacterium increases, *C. albicans* becomes pathogenic and is responsible for early childhood caries [3] and painful mucosal infections [4, 5]. It has been generally acknowledged that the biofilm formation of *C. albicans* plays an important role in pathogenesis [6]. What is more, the severity is largely associated with morphological transition between yeast and hyphae [7, 8], and the latter affects in invasion to targeted tissue such as epithelium in oral [7].

Accelerating the demineralization of enamel surfaces, *Streptococcus mutans* (*S. mutans*) is one of the major pathogens in dental caries [9]. Several studies have indicated *S. mutans* and *C. albicans* may interact with each other and form dual-species biofilms [10–12]. Several in vitro studies found that *C. albican*s/*S. mutans* dual-species biofilms reach higher cell numbers than single-species biofilms [13], and some in vivo studies demonstrated that severity of lesions in the dually infected animals showed a dramatic increase comparing with singly infected ones [14, 15]. However, other researchers reported that some subproducts of *S. mutans* inhibited the biofilm formation, morphogenesis, and pathogenicity of *C. albicans* [16]. In conclusion, the relationship between *C. albicans* and *S. mutans* in dual-species biofilms is still controversial.

In recent years, with the extensive usage of broad-spectrum antibiotics and implanted medical materials, the incidence of candidiasis has been increased nearly 40-fold [17]. Natural products and medicine have become a hot spot in the whole world with the advantages of being safe, reliable, long-lasting efficacy, few side effects, and so on. They are more suitable for anti-infective treatment in special populations, such as the toddler, the older, and the HIV patients [18]. Currently, many cariostatic natural products have been identified, typical natural products such as ginkgoneolic acid [19], epigallocatechin gallate [20], cranberry polyphenols [21], and Chenopodium ambrosioides [22]. When it comes to antifungal treatment, essential oils (EOs) of two Moroccan endemic thymes (Thymus broussonetii and T maroccanus) have been identified with anticandidal activity [23] and synergism with amphotericin B and fluconazole [24]. LongZhang Gargle was an important product created from Chinese folk herbs and used firstly by Miao people, an ethnic group in Southwest China. It has been manufactured by Guiyang Xintian Pharmaceutical Company, Guizhou, China, for nearly 20 years. At present, LongZhang Gargle is mainly applied for treating gingivitis, periodontitis, and oral ulcer in clinical treatment [25]. The root and leaves of *Toddalia asiatica* (L.) Lam., Cortex Lycii, and *Cimicifuga foetida* were the main components present in the gargle. As the main functional ingredient, *Toddalia asiatica* (L.) Lam. (*T. asiatica*), a woody liana, which belongs to the family Rutaceae, is a medical plant traditionally used to treat coughs, fevers, and various diseases. All parts of the plant are claimed to have medicinal value, and coumarin and alkaloids are the major bioactive constituents of it, playing an important role in various biological activities such as anti-inflammatory, analgesic, antibacterial and antitumour. Currently, the antibacterial and antifungal activities of T. asiatica have been demonstrated in planktonic growth of some microorganism, such as *Staphylococcus aureus*, *Staphylococcus epidermidis*, *Escherichia coli*, *Pseudomonas aeruginosa*, *Klebsiella pneumoniae*, and *Candida albicans* [26–28]. A study also justified that LongZhang Gargle suppresses not only planktonic growth of *S. mutans* but also biofilm formation and acid production, suggesting that the prospect of LongZhang Gargle as an anticariogenic treatment in vitro [29]. Confined with clinical value in curing oral disease of LongZhang Gargle, previous studies refer little to the pharmacological mechanism of it. In addition, the existing researches of *T. asiatica* ignore the effect on oral *C. albicans* biofilms and dual-species biofilms.

At present, there is no report about the effect of LongZhang Gargle on the formation of dual-species *C. albicans*/*S. mutans* biofilms, and their relationship also needs further study. Since biofilms are the main pathogenic factor of oral microorganisms [30], we developed single and dual-species biofilm models to study biofilm biomass, biofilm activity, biofilm structure, synthesis of extracellular polysaccharides (EPS), and changes of hyphae of *C. albicans* in the presence of physiologically relevant concentrations of LongZhang Gargle.

## 2. Materials and Methods

### 2.1. Chemicals and Bacterial and Fungal Strains and Growth Conditions

The agent of LongZhang Gargle was provided by Guiyang Xintian Pharmaceutical Co. Ltd., Guizhou, China. *S. mutans* UA159 (ATCC 700610) and *C. albicans* strains SC5314 (ATCC 10691) were provided by State Key Laboratory of Oral Diseases, Sichuan University, Chengdu, China. *C. albicans* was grown in YPD medium (1% yeast extract, 2% peptone, and 2% D-glucose) at 37°C anaerobically with 5% CO_2_ [31]. *S. mutans* was grown in brain-heart infusion (BHI) medium at 37°C anaerobically with 5% CO_2_ [32]. YNBB (0.67% YNB, 75 mM Na_2_HPO_4_-NaH_2_PO_4_, 2.5 mM N-acetylglucosamine, 0.2% casamino acids, and 0.5% sucrose) was used to support the growth of single and dual-species biofilm of *S. mutans* and *C. albicans* [13]. For the dual-species mixture, we took 2 × 10^4^ colony-form units (CFU)/mL of *C. albicans* and 2 × 10^6^ CFU/mL of *S. mutans* [14]. Alexa Fluor 647 (Invitrogen, Carlsbad, CA, USA) was a labelled dextran conjugate, as a red fluorescent stain for EPS. SYTO 9 (Molecular Probes, Eugene, OR, USA) was a green fluorescent for nucleic acid stain.

### 2.2. Minimum Inhibitory Concentration (MIC) and Minimum Bactericidal Concentration (MBC)

The MIC and MBC of LongZhang Gargle against *C. albicans* were determined by double dilution method modified from that of Lombardo Bedran et al. [33]. Each well containing 180 *μ*L of serially diluted LongZhang Gargle (2%, 4%, 8%, 16%, 32%, and 64%) in YPD culture media was inoculated with 20 *μ*L *C. albican*s fluid for an overnight culture, diluted in fresh YPD culture media to obtain an OD_600nm_ of 0.2 (about 10^8^ CFU/mL) in a 96-well tissue culture plate. There are three parallel samples in each concentration of group, and YPD without gargle was used as a control. The MIC was the lowest concentration of the gargle that no bacteria grown in the broth. To determine the MBC, the 200 *μ*L cultures at a drug concentration above MIC were inoculated into YPD agar plates and incubated at 37°C for 24 h. The MBC was the lowest concentration of the gargle that no fungus could be observed on the agar plate.

### 2.3. Biofilm Formation

Cultures of *C. albicans* and *S. mutans* from single colonies were incubated overnight and adjusted to a concentration of 2 × 10^5^ CFU/mL (*C. albicans*) and 2 × 10^7^ CFU/mL (*S. mutans*). Suspensions (20 *μ*L) of *C. albicans* and 180 *μ*L YNBB medium with different concentrations of LongZhang Gargle below the MIC were incubated into 96-well microtiter plates to form single-species biofilms. Equal volumes of each strain (10 *μ*L) and 180 *μ*L YNBB medium with different concentrations of LongZhang Gargle below the MIC were incubated into 96-well microtiter plates for the formation of dual-species biofilms. Suspensions (200 *μ*L) of *C. albicans* and 1.8 mL YNBB medium with different concentrations of LongZhang Gargle below the MIC were also incubated in 24-well microtiter plates for single-species biofilm formation. Equal volumes of each strain (100 *μ*L) and 1.8 mL YNBB medium with different concentrations of LongZhang Gargle below the MIC were incubated into 96-well microtiter plates for the formation of dual-species biofilms. Glass coverslips (5 mm in diameter) were prefixed in each well in 24-well microtiter plates. The plates were incubated at 37°C anaerobically with 5% CO_2_ for 24 h.

### 2.4. Biofilm Biomass Assay by Crystal Violet Staining

The effect of LongZhang Gargle on single-species biofilm of *C. albicans* and dual-species biofilm of *C. albicans* and *S. mutans* biofilm formation was measured by the method modified from that of Li et al. [34] and Assaf et al. [35]. After incubated in a 96-well microtiter plate for 24 h, the biofilm in each well was gently washed three times with phosphate-buffered saline (PBS), fixed with 95% methanol for 15 min, washed with PBS, stained with 0.5% crystal violet for 30 min, and then washed three times with PBS to remove the unbound crystal violet. After that, 200 *μ*L of 100% ethanol was added to each well to dissolve the crystal violet on the biofilm for 1 hour, and the extract was read at 595 nm in a spectrophotometer [36]. The percentage of inhibition was calculated using the following formula: (1 − *A*_595_ of the test group/*A*_595_ of blank control) × 100%.

### 2.5. Cell Activity Assay of Biofilm

Colorimetric method such as MTT assay was used to evaluate the cell activity effect of LongZhang Gargle on single-species biofilm of *C. albicans* and dual-species biofilm of *C. albicans* and *S. mutans*. After incubated in a 96-well microtiter plate for 24 h, the biofilm in each well was gently washed three times with PBS, stained with 0.5 mg/mL MTT (sterilized by 0.22 *μ*m size pore filter), protected from light at 37°C for 1 hour, and then 100 *μ*L DMSO was added to each well. 96-well microtiter plate was kept shocking and protected from light at 37°C for 1 hour. After that, the MTT-DMSO mixture was transferred into a new 96-well microtiter plate to read at 570 nm in a spectrophotometer.

### 2.6. Morphology of Biofilms by Scanning Electron Microscopy

After incubation in 24-well microtiter plates for 24 h, the biofilm-coated glass coverslips were gently washed with PBS, immersed in 2.5% glutaraldehyde at 4°C overnight, and washed with PBS. Biofilms were then dehydrated by a series of different concentrations of ethanol (30, 50, 70, 80, 85, 90, and 95%), immersed for 10 min in 100% ethanol, and dried in a desiccator [29]. After sputter coating with gold-palladium, samples were analyzed in a scanning electron microscope at 2000×, 5000×, and 10,000× magnification.

### 2.7. Confocal Laser Scanning Microscopy of Cell and EPS in Mixed Biofilms

Biofilms were observed for the volume of their major components (fungus in single-species biofilm, EPS, and bacteria in dual-species biofilm) by confocal laser scanning microscopy modified from Xiao and Koo [37] and Qiu et al. [32]. 2.5 *μ*M Alexa Fluor 647 dextran conjugate labeling EPS was added to each well during the formation of biofilms and protected from light. After incubation for 24 h, biofilms were gently washed with PBS and incubated with 1 *μ*M SYTO 9 fluorescent dye for 20 min in the dark. Biofilms were then washed with PBS and dried. ProLong gold antifade reagent was added to the biofilms, and images were obtained by confocal laser scanning microscope (CLSM) [37] under 63x oil immersion objectives. The mean fluorescence intensity was evaluated by Image J 6.0 software.

## 3. Results

### 3.1. LongZhang Gargle Exhibited Antimicrobial Activity against *C. albicans*

The antimicrobial effect of LongZhang Gargle was determined by measuring MIC and MBC against *C. albicans*. The results showed that LongZhang Gargle inhibited the growth of planktonic *C. albicans* at MIC of 16% and MBC of 32%. Considering the MIC of LongZhang Gargle against *S. mutans*, which has been reported before, we chose 2%, 4%, and 8% of LongZhang Gargle in the present study.

### 3.2. LongZhang Gargle Inhibited Biomass of Single-Species Biofilm of *C. albicans* and Dual-Species Biofilms of *C. albicans* and *S. mutans*

**Figure 1:**
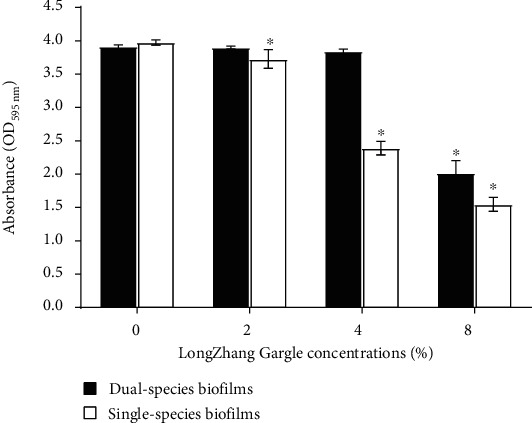
Biofilm biomass of single-species and dual-species biofilms at varying LongZhang Gargle concentrations (0, 2, 4, and 8%) at OD 595 nm. The white bars indicate *C. albicans*, and the black bars indicate dual-species of *S. mutans* and *C. albicans*. Asterisks indicate the statistical differences compared to the 0% LongZhang Gargle. The error bars indicate the standard deviation (SD).^∗^*P* < 0.05.

The effect of LongZhang Gargle on biofilm formation was determined by measuring the absorbance at 595 nm in crystal violet assay. As is shown in Figure 1, single and dual-species biofilms biomass was slightly inhibited in the presence of LongZhang Gargle. LongZhang Gargle significantly inhibited single-species biofilms formation at 2% (*P* < 0.05), 4% (*P* < 0.05), and 8% (*P* < 0.01). Furthermore, differences are significant between 2% and 4% (*P* < 0.05) and 4% and 8% (*P* < 0.05). This demonstrated that the inhibitory effect was dosage-dependent. However, for dual-species biofilms of *C. albicans* and *S. mutans*, statistical analysis indicated that dual-species biofilms were significantly inhibited when LongZhang Gargle at 8% (*P* < 0.05) but no significant difference was found between control (0%), 2%, and 4%. It suggested that only a higher concentration of LongZhang Gargle (8%) could inhibit dual-species biofilms formation, while lower concentrations of LongZhang Gargle (2%, 4%) had no obvious effect on biofilm formation when it referred to dual-species. The inhibition percentages are 39.89% and 62.21% at 4% and 8% concentration of LongZhang Gargle. 8% was MBIC 50, the minimum concentration that resulted in at least 50% inhibition of biofilm formation, comparing with the control group.

### 3.3. LongZhang Gargle Suppressed Cell Activity of Single-Species Biofilm of *C. albicans* and Dual-Species Biofilms of *C. albicans* and *S. mutans*

**Figure 2:**
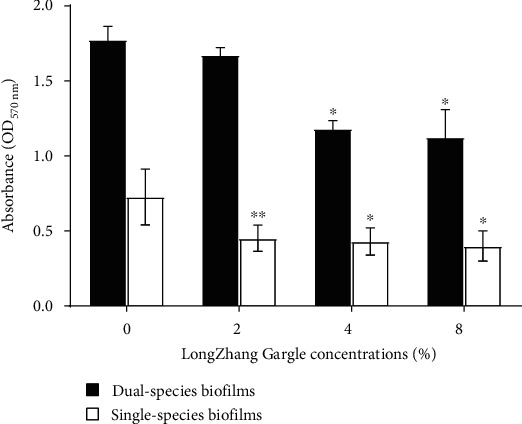
Cell activity of single-species and dual-species biofilms at varying LongZhang Gargle concentrations (0, 2, 4, and 8%) at OD 570 nm. The white bars indicate *C. albicans*, and the black bars indicate dual-species of *S. mutans* and *C. albicans*. Asterisks indicate the statistical differences compared to the 0% LongZhang Gargle. The error bars indicate the standard deviation (SD). ^∗^*P* < 0.05.

The effect of LongZhang Gargle on cell activity of biofilm was determined by measuring the absorbance at 570 nm in MTT assay. As is shown in Figure 2, cell activity in both single- and dual-species biofilms was slightly suppressed in the presence of LongZhang Gargle. LongZhang Gargle significantly suppressed cell activity in single-species biofilm formation at 2% (*P* < 0.05), 4% (*P* < 0.05), and 8% (*P* < 0.05). For dual-species biofilms of *C. albicans* and *S. mutans*, 4% and 8% concentration of LongZhang Gargle suppressed cell activity, but no significant difference was found between control (0%) and 2%.

### 3.4. The Alteration of Morphology in Single-Species Biofilm of *C. albicans* and Dual-Species Biofilms of *C. albicans* and *S. mutans*

**Figure 3:**
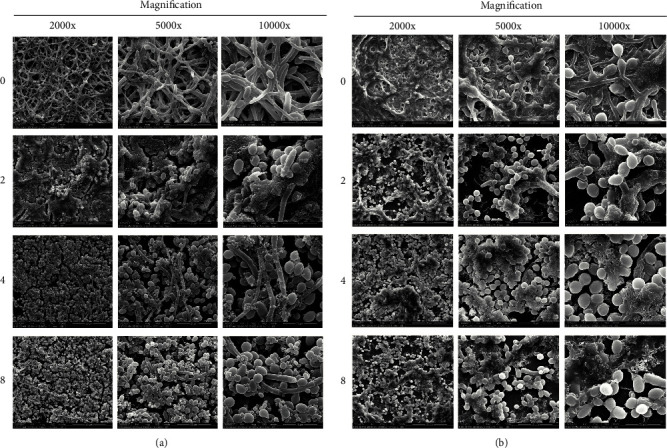
Scanning electron microscopy images of different biofilms. (a) Morphology of single-species biofilms of *C. albicans* treated with 0, 2, 4, and 8% of LongZhang Gargle for 24 h in YNBB broth. Magnification was 2000×, 5000×, 10000×, and 20000×, respectively, for each concentration. (b) Morphology of dual-species biofilms of *C. albicans* and *S. mutans* treated with 0, 2, 4, and 8% of LongZhang Gargle for 24 h in YNBB broth. Magnification was 2000×, 5000×, 10000×, and 20000×, respectively, for each concentration.

Scanning electron micrographs displayed the distribution of yeast and filamentous forms of *C. albicans* in single-species biofilm (shown in Figure 3(a)) and *S. mutans* and *C. albicans* cells in the dual-species biofilms (shown in Figure 3(b)). In single-species biofilm of *C. albicans*, the control group (without the gargle) witnessed a high density of *C. albicans*. With the concentration of LongZhang Gargle increasing, the biomass and thickness of biofilm were decreased gradually. Hyphae of *C. albicans* were reduced gradually and dramatically, and the ratio of hyphae and fungal spores fell down. In dual-species of biofilms, *S. mutans* cells were frequently observed adhering to the hyphae of *C. albicans*. Compared with the single-species biofilms, the dual-species biofilms reached higher biomass and more cell in the same concentration. With the concentration of LongZhang Gargle increasing, the alteration of *C. albicans* was the same as that in single-species biofilm, and *S. mutans* cells in biofilm were reduced gradually. In addition, biofilm integrity was disrupted when treated with different concentrations of LongZhang Gargle. Cell aggregates became sparse in the presence of 8% LongZhang Gargle, which was compact in control groups, while in higher concentration of LongZhang Gargle, more scattered bacteria could be seen.

### 3.5. LongZhang Gargle Inhibited Cell Numbers, Hyphae of *C. albicans*, and EPS Production in Biofilm

**Figure 4:**
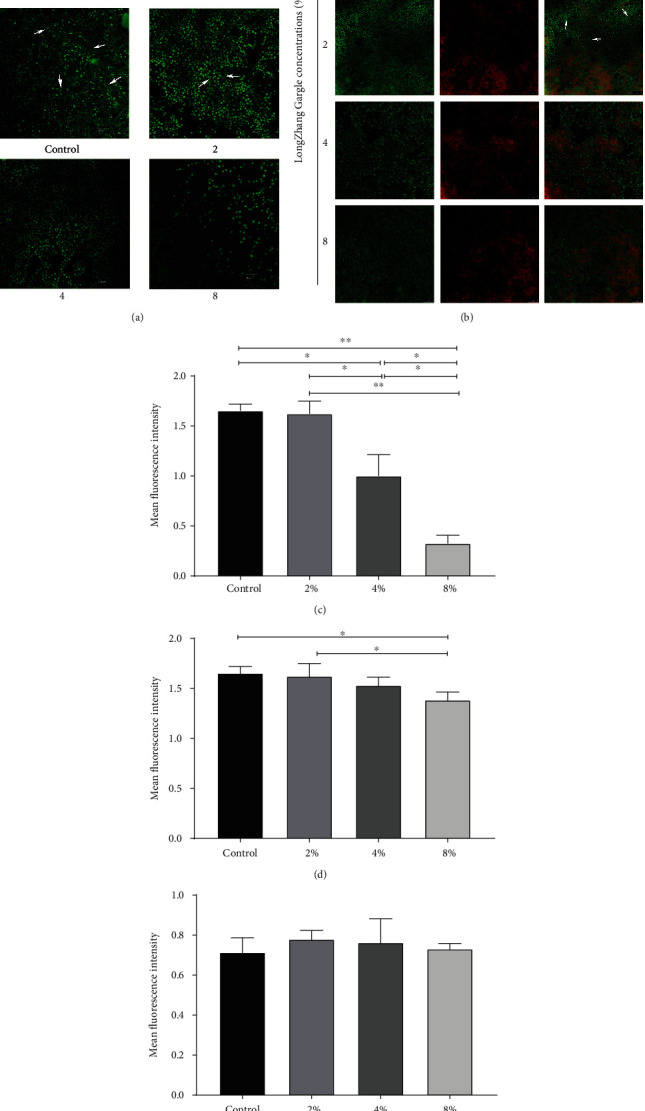
Confocal laser scanning microscopy images of different biofilms. (a) Images of single-species biofilms of *C. albicans* treated with 0, 2, 4, and 8% of LongZhang Gargle for 24 h. Cells were labelled green (SYTO 9), EPS was labelled red (Alexa Fluor 647), and red and green superimposed appear as yellow. Magnification was 63× for oil immersion objective. (b) Images of dual-species biofilms of *C. albicans* and *S. mutans* treated with 0, 2, 4, and 8% of LongZhang Gargle for 24 h. Cells were labelled green (SYTO 9), EPS was labelled red (Alexa Fluor 647), and red and green superimposed appear as yellow. Magnification was 63× for the oil immersion objective. (c) Mean fluorescence intensity of single-species biofilms at different concentration of LongZhang Gargle. (d) Mean fluorescence intensity of double-species biofilms at different concentrations of LongZhang Gargle. (e) Mean fluorescence intensity of EPS in double-species biofilms at different concentrations of LongZhang Gargle. All values on three duplicate samples were presented as mean with SD (^∗^*P* ≤ 0.05; ^∗∗^*P* ≤ 0.01).

Hyphae of *C. albicans* and EPS of *S. mutans* both play the key role in biofilm formation, suggesting a material basis for the development of oral diseases. We conducted the CLSM study to investigate how these components were affected by LongZhang Gargle (shown in Figure 4). In a single-species biofilm of *C. albicans* (shown in Figure 4(a)), the control group (without the gargle) witnessed a high density of *C. albicans*, especially filamentous forms. With the concentration of LongZhang Gargle increasing, the structure of biofilm became loose. Fungus and hyphae of *C. albicans* decreased gradually, meanwhile, the proportion of hyphae to fungal spores fell down. All the result can be seen clearly as hyphae, and fungal spores have been labelled in green fluorescence. Because *C. albicans* does not produce EPS, it is rare to observe EPS staining red in the field of vision. In dual-species biofilm (shown in Figure 4(b)), we can see the cells of *C. albicans* and *S. mutans* (green) in different size and shape, and the distribution of EPS (red) is consistent with *S. mutans*. With the concentration of LongZhang Gargle increasing, the bacteria of *S. mutans* decreased gradually, and the alterations of *C. albicans* were the same as it in single-species biofilm. However, EPS in dual-species biofilms did not reduce obviously.

## 4. Discussion

Bacteria and fungi have been verified to symbiosis in the human body, and their common biofilms play an important role in several diseases in oral [15]. Their interactions may influence the transition from healthy to sick state within a specific host niche [38]. The human body is a good habitat for numerous microorganisms [1], and more than 700 different common species have been identified in oral cavity over the past few decades [39]. *S. mutans* and *C. albicans* are typical bacteria and fungi, which can form biofilm with a special structure on teeth [9].

Many studies have proved that the active ingredients of traditional Chinese medicine can effectively inhibit the growth of bacteria and fungi [28]. At the same time, recent clinical trials have proved that the combination of Chinese and Western medicine greatly increased the treatment efficacy of oral infections [23]. However, as one of the traditional Chinese medicine, pharmacological effects of LongZhang Gargle have never been studied very clearly. In the present study, we demonstrated the activities of LongZhang Gargle to inhibit the planktonic growth of *C. albicans*. Diluted LongZhang Gargle at a concentration of 16% could effectively and completely inhibit the growth of *C. albicans*, and at the concentration of 32% showed an obvious bactericidal activity. Because dark color of excessive-high concentration of LongZhang Gargle could affect the experimental observation, so we chose 8%, 4%, and 2% concentrations of LongZhang Gargle in our study. However, the fungus in the human mouth exists in biofilms rather than planktonic form, and other oral microorganism species in biofilms probably also contribute to oral diseases. Therefore, we made two kinds of model in single-species and dual-species biofilm and explored the effects of LongZhang Gargle on *S. mutans* and *C. albicans* biofilm formation as described above. Our study showed that both in single and dual-species biofilms the application of LongZhang Gargle can inhibit biomass of biofilm (Figures 1 and 3, amount of green fluorescent for cell in Figure 4), suppressed cell activity in biofilm (Figure 2), and lead to changes of biofilm structure (shape of hyphae in Figures 3 and 4). For further study, other common fungus, bacteria, and their biofilms could also be similarly investigated.

Biofilms provide protection against antibiotics and supply a barrier to prevent or reduce the penetration of antimicrobial agents through the matrix, so they are also considered as an initiating factor for dental diseases [40, 41]. Therefore, the reduction of stable biofilm formation is an effective way to prevent infection. In this study, we identified that the dual-species biofilms of *S. mutans* and *C. albicans* were susceptible to LongZhang Gargle. We found that single-species biofilm formation was significantly suppressed at the concentration of 2% LongZhang Gargle, and dual-species biofilm formation was significantly suppressed at the concentration of 8%. Moreover, we also found that the MBIC 50 (8%) was lower than MIC (16%), which indicated that the gargle was more effective to inhibit aggregation of cells and reduce biofilm formation compared with its antifungal and antibacterial activity. The principle of the MTT assay method is measuring the activity of mitochondria in *C. albicans* [42, 43], which is related to the formation of hyphae [7]. In addition, we observed that the biofilm was relatively thick and tight without the gargle compared with those samples treated with gargle at different concentrations. As the concentration of the gargle increases, the biofilm integrity and structure is gradually being disrupted, Moreover, this disruption to biofilm integrity was also dosage-dependent. At a concentration of 8%, biofilm became very sparse. Furthermore, we found that the volume of hyphae form declined sharply when adding the gargle, even though the concentration was relatively low.

Many documents have confirmed that hyphae are important virulence factors of *C. albicans* [44]. Hyphae forms of *C. albicans* have stronger pathogenicity than yeast forms and play an important role in the invasion of epithelial cells and oral tissue destruction [7]. Therefore, inhibiting the expression of virulence of mycelium is an important target for the inhibition of the biofilms formation, the prevention, and treatment of *C. albicans* infection [8]. The inhibitive effect of LongZhang Gargle on expression of hyphae forms has been justified in the present study. At the meantime, morphological changes of *C. albicans* can be caused by not only growth environment impact, such as growth temperature, serum, neutral pH, and high concentrations of carbon dioxide, but also other microbe that resided in common biofilms [7]. In our study, pH of several concentrations of LongZhang Gargle can be considered the same with it of the control group (supplementary data Figure S1). We found that with the increase of the concentration of LongZhang Gargle, the phase of fungus changed a lot, such as thedecrease of hyphae forms of *C. albicans* in biofilms (Figures 4(a) and 4(b)). What is more, LongZhang Gargle also had the same effect in the dual-specie biofilms formed by *C. albicans* and *S. mutans*. The results show that LongZhang Gargle has not only the previously reported inhibitive effect to *S. mutans* and acid-producing effect but also suppressive capacity to fungus, as well as fungal and bacterial dual-species biofilm, which is little previously reported before. The cause of this phenomenon may be as follows: first is the inhibitory effect of LongZhang Gargle to hyphae of *C. albicans*; second, the application of LongZhang Gargle is also able to break the original interaction between bacteria (such as *S. mutans* in present experiment) and *C. albicans*, especially autoagglutination and synergistic relationship between them. With the reduction of mycelium of *C. albicans*, we can also observed a reduction of the amount of *S. mutans*, which is adhered on mycelia of *C. albicans*, but there mostly are no changes in the synthesis of EPS (Figure 4(b)). This phenomenon can be explained by the irritability of *S. mutans*, which is activated in a relatively inferior environment. Because of irritability, each survival bacteria of *S. mutans* under LongZhang Gargle relatively produced more EPS than in the control group, so even the amount of *S. mutans* reduced, and the total amount of EPS did not changed a lot.

It has confirmed that the natural substances extracted from Chinese medicine do have antimicrobial effect in oral infection of *C. albicans*. Due to better treatment efficacy and few side effects, Chinese medicine has become the focus of study. Our experiments confirmed that LongZhang Gargle can be used as a future antibacterial and antifungal drug due to its ability to inhibit the formation of filaments, biofilms, and cell activity in biofilms. At the same time, considering less side effects and long-last efficacy, we speculate that LongZhang Gargle can be used in prevention for oral infection and will be an integrated and basic drug to prevent and cure the common oral diseases including dental caries, gingivitis, periodontal disease, and oral ulcer. To confirm our speculation, the mechanism such as gene expression in biofilm and host cell need further investigation, and clinical studies of LongZhang Gargle need to be conducted.

## 5. Conclusions

LongZhang Gargle showed antimicrobial effect on single-species biofilms of *C. albicans* and dual-species biofilms of *C. albicans* and *S. mutans* in vitro, which may indicate its potential use as an oral antimicrobial agent in the treatment of *C. albicans* and *S. mutans* oral diseases.

## Data Availability

The full data used to support the findings of this study are available from the corresponding author upon request.

## Supplementary Materials

Supplementary Materials Figure S1: the influence of LongZhang Gargle on pH in growth environment of biofilms. The error bars indicate the standard deviation (SD). There is no statistical significance from control.
