# Supplementary material for: Effect of LongZhang Gargle on Dual-Species Biofilm of Candida albicans and Streptococcus mutans
Source: Biomed Res Int. 2021 Mar 22;2021:6654793. doi: 10.1155/2021/6654793 (PMC8007335; doi:10.1155/2021/6654793)

Supplementary Materials

Figure S1: The influence of LongZhang Gargle on pH in growth environment of biofilms. The error bars indicate the standard deviation (SD). There is no statistical significance from control.

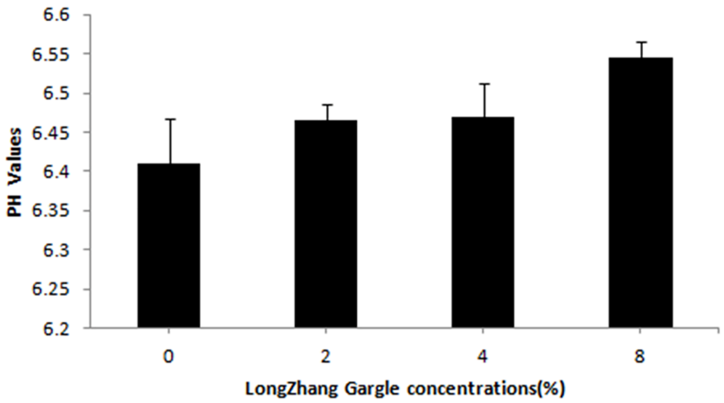

Supplement: Supplementary Materials — Figure S1: the influence of LongZhang Gargle on pH in growth environment of biofilms. The error bars indicate the standard deviation (SD). There is no statistical significance from control. [file 6654793.f1.pdf]
